# A systematic review and meta-analysis of randomized controlled trials of palliative care for pain among Chinese adults with cancer

**DOI:** 10.1186/s12904-019-0456-z

**Published:** 2019-08-08

**Authors:** Xin-Xin Zhao, Meng Cui, Yi-Hang Geng, Yi-Long Yang

**Affiliations:** 1 Hospice Ward, Shengjing Hospital of China Medical University, Shenyang, China; 2 School of Public Health, China Medical University, Shenyang, China; 3 Department of Social Medicine, School of Public Health, China Medical University, No.77 Puhe Road, Shenyang North New Area, Shenyang, Liaoning 110122 People’s Republic of China

**Keywords:** Pain, Palliative care, Chinese adults with cancer, Meta-analysis

## Abstract

**Background:**

Pain is one of the most common symptoms that has a severe impact on quality of life and is associated with numerous psychosocial issues in cancer patients. Palliative care, which is a recent development in China, mainly focuses on symptom control and provides psychosocial support in order to improve quality of life for terminally ill patients. This meta-analysis aimed to evaluate the effects of palliative care on cancer pain in China.

**Methods:**

The four most comprehensive Chinese academic databases-CNKI, Wanfang, Vip and CBM-were searched from their inception until July 2019. Medline/PubMed, Web of Science, EBSCO and internet search (Google and Google Scholar) were also searched. Randomized controlled studies assessing the effects of palliative care on cancer pain were analyzed. The pooled random-effect estimates of standardized mean difference (SMD) and 95% confidence intervals (CI) were calculated. Subgroup analysis was conducted by moderating factors for heterogeneity.

**Results:**

The present meta-analysis included 18 studies with a total of 1370 patients. The random-effect model showed a significant effect size of palliative care on cancer pain (SMD = 1.475, *p* < 0.001; 95% CI = 1.071–1.878). Age, pharmacological/non-pharmacological strategies and publication date could account for the heterogeneity through subgroup analysis to some extent.

**Conclusions:**

Palliative care was largely effective for relieving pain among Chinese adults with cancer, indicating that an adequate system should be urgently established to provide palliative care for cancer patients in Chinese medical settings. However, given the extent of heterogeneity, our findings should be interpreted cautiously.

**Electronic supplementary material:**

The online version of this article (10.1186/s12904-019-0456-z) contains supplementary material, which is available to authorized users.

## Background

Pain is one of the most common and distressing symptoms in cancer patients, either because of the disease itself or the related treatments. The prevalence rate of pain was 50.7% in all cancer stages, and 66.4% in advanced stage; moderate to severe pain was reported by 38.0% of all patients, and by 51.9% in advanced stage [1]. Cancer pain could impair patients’ quality of life [2] and was associated with numerous psychosocial distresses [3]. Unfortunately, approximately 33% of untreated cancer pain have been amply documented [4], and thus pain control remains a core issue in cancer patient care.

Contrary to traditional oncologic care, advanced cancer patients received the treatment with palliative intent and the main emphasis placed on palliative care (PC) in some settings [5]. Considered as an interdisciplinary care, PC focuses on symptom control and provides psychosocial and other support (e.g., decision making) in order to improve quality of life for persons with serious illness [6]. Although pain control plays a central role in managing patients’ distress under the process of PC, not much is generally known regarding the effects of PC on cancer pain.

Due to multi-factorial etiology of cancer pain, the related treatment should be based upon the bio-psycho-social approach [7]. Compared to the traditional pain-control methods, PC considers not only biomedical factors but also patients’ psychosocial and spiritual distress [6]. However, relatively few studies have been carried out to explore the effects of PC on cancer pain. The randomized controlled trials (RCTs) of PC paid more attention to the evaluation of quality of life, symptom burden and psychological distress [8–10], and systematic reviews mainly focused on the prevalence rate and severity of cancer pain [1, 11]. To the best of our knowledge, few meta-analysis of RCTs have evaluated the effects of PC on cancer pain.

Among Chinese cancer patients, conducting such meta-analysis is vitally important for the following reasons. The first reason is attributed to the large number of Chinese cancer patients. The latest data revealed that the numbers of new cases and deaths were 4.292 million and 2.814 million, which accounted for 21.8 and 26.9% of global cancer population, respectively [12]; second, the development of PC originated in Western countries is in the initial stage in China, it is of importance to explore whether PC could effectively alleviate cancer pain; third, several RCTs of PC on cancer pain have been published in Chinese journals, but there has not been a comprehensive meta-analysis to review these literatures and assess the effects of PC; finally, Chinese researchers paid more attention to traditional Chinese medicine [13–15] rather than PC for reducing cancer pain, which could neglect the importance of PC to some extent.

The aim of this meta-analysis was to perform a systematic review and evaluate the effect of PC on reducing cancer pain reported by RCTs in an attempt to obtain accurate profile of cancer pain under PC in China.

## Methods

### Literature search

A systematic search was conducted to identify published literatures about the effect of PC on cancer pain in China. The CNKI (China National Knowledge Infrastructure), Wanfang, Vip and CBM (Chinese Biomedical Literature Database), which are the four most comprehensive Chinese academic databases, were searched from their inception until July 2019. The search was constructed and performed by a professional medical librarian. In order to expand searches, Medline/PubMed, Web of Science (SCIE) and CINAHL (EBSCO) were also searched from their inception until July 2019 without language restrictions. Search strategies for domestic and international databases were shown in Additional file 1. The reference lists of relevant articles obtained were also screened. Additionally, the search keywords used for Google and Google Scholar search were: cancer, China and randomized controlled trials, in combination with palliative care, hospice care or terminal care. The screening of the abstracts/titles and full-text articles were performed twice by two authors (XXZ and MC) independently to reduce reviewer bias.

### Inclusion and exclusion criteria

We included all studies in which: (1) subjects age ≥ 18 years; (2) RCTs; (3) subjects were diagnosed with cancer; (4) pain was evaluated by well-validated measures. We excluded studies in which: (1) PC was not described in details, which could not provide valuable leads for further research to conduct PC; (2) studies focused on the separate components of PC, such as psychosocial support, pain control or spiritual care; (3) studies did not provide the post-test score of cancer pain. Eligibility judgment and data extraction were recorded and carried out independently by two authors (YHG and XXZ) in a standardized manner. Any disagreements were resolved by discussion and the involvement of another author (YLY).

### Quality assessment

The modified Jadad scale for assessing quality of RCTs was adopted in this study [16], which has been used in our previous meta-analysis [17]. The modified Jadad scale is an eight-item scale designed to assess randomization, blinding, withdrawals/dropouts, inclusion/exclusion criteria, adverse effects, and statistical analysis. In this meta-analysis, blinding (2 points) and adverse effects (1 point) were excluded due to the characteristics and effects of PC. Thus, the score for each study ranged from 0 (lowest quality) to 5 (highest quality). We defined three categories: the study was considered to have high quality (low risk of bias) if it scored 4 points or above, studies that scored 1 point or below were categorized as having low quality (high risk of bias), studies that scored 2 or 3 points were considered as having medium quality (moderate risk of bias). Any disagreements with authors (XXZ and MC) were resolved by discussion and the involvement of another author (YLY).

### Data extraction

We used the Endnote to do the screening, and employed the Excel to do the data extraction, which is a standardized data abstraction form designed to capture and code all relevant study-level information required for analysis. For the included studies, two authors independently extracted data (YHG and MC). Disagreements were resolved by discussion and the involvement of another author (YLY). Extracted data included author name, year of publication, age range and mean age, simple size, assessment instruments, cancer type, pharmacological/non-pharmacological strategies, care time, settings and the values of mean and standard deviation (SD) in experimental-control group.

Pain control in PC mainly included both pharmacological and non-pharmacological strategies. Analgesic and adjuvant drugs are the most commonly used pharmacological strategies, which were endorsed and promoted by World Health Organization (WHO) in the now-famous ‘analgesic ladder’ for managing cancer pain properly. Non-pharmacological strategies included several bio-psycho-social approaches, such as music therapy, psychological interventions (e.g., relaxation), traditional Chinese medicine (e.g., herbal, acupuncture, moxibustion, massage) and others. Data could not be extracted when psychological interventions mainly focused on psychological distress rather than cancer pain.

### Meta-analysis

#### Assessment of overall effect size

We computed the effect size of standardized mean difference (SMD) for each study by subtracting the average post-test score of the control group from that of the experimental group and dividing the result by the pooled SDs of the experimental-control group. Means and SDs of cancer pain were used for computation of SMD (Cohen’s d). A SMD of 1 indicates a relatively stronger improvement in experimental group by one standard deviation larger than the mean of control group. According to the expected heterogeneity across studies, the pooled random-effect estimates of SMD and 95% confidence intervals (CI) were used as the summary measure of effect size. Cohen’s criteria were used to interpret the magnitude of SMD [18]: a value of 0.20–0.50 corresponds to small effect sizes, 0.50–0.80 to medium and a value over 0.80 to large effect sizes, which is also employed and supported by other studies [19–21]. A two-tailed *P* value of < 0.05 was considered to be significant. Overall effects and other statistical analyses were analyzed using the statistical software Stata 11.0.

#### Assessment of heterogeneity

Heterogeneity was evaluated with the Q statistic and I^2^ statistic. The Q statistic is used to assess whether differences in results are compatible with chance alone, but it is sensitive to the number of studies [22]. The I^2^ statistic which denotes the variance among studies as a proportion of the total variance was also calculated and reported, because I^2^ statistic is not sensitive to the number of studies [22]. Larger values of I^2^ show increasing heterogeneity. An I^2^ of 0% shows no observed heterogeneity, while 25% shows low, 50% moderate, and 75% high level of heterogeneity [23].

#### Subgroup analysis

When the hypothesis of homogeneity was rejected by the Q statistic and I^2^ statistic, subgroup analysis was conducted to explore the potential moderating factors for heterogeneity. For subgroup analyses, the heterogeneity within groups was tested. In this meta-analysis, subgroup analysis was conducted for moderating factors, including age, cancer type, pharmacological strategies (experimental-control group), non-pharmacological strategies in the experimental group and publication date. If subgroup analysis restricted to studies of these factors could reduce the variance (i.e., Q statistic and I^2^ statistic), and these moderating factors were indeed behind the heterogeneity observed.

## Results

### Study selection

**Fig. 1:**
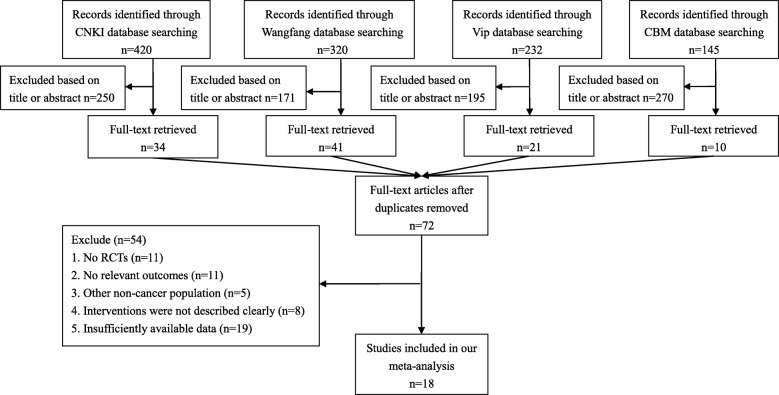
Selection process of studies for the meta-analysis (Chinese databases). Abbreviations: RCTs, randomized controlled trials; CNKI, China National Knowledge Infrastructure; CBM, Chinese Biomedical Literature Database

The total number of included studies was 18 in the present study. As shown in Fig. 1, we identified the eligible articles through the database of CNKI (*n* = 420), Wangfang (*n* = 320), Vip (*n* = 232) and CBM (*n* = 145). The titles and abstracts of these articles were respectively reviewed by the three authors (MC, YHG, YLY), and the full-text articles without duplicates (*n* = 72) were selected for further examination. Based on these 72 studies, 54 did not meet the inclusion criteria. In total, 18 RCTs about the effect of PC on cancer pain were included [24–41].

In order to expand searches (see Additional file 2), we also searched the international databases of Medline/PubMed (*n* = 32), SCIE (*n* = 22) and EBSCO (*n* = 3), but there were no studies that met our inclusion criteria.

### Characteristics of included studies

Study characteristics were listed in Table 1. The included studies published from 2006 to 2019 comprised 1370 subjects (experimental group = 705, control group = 665) with mean age of 62.07 years (median: 61.31; range: 43–76.26). Cancer pain was assessed by single-item unidimensional tools, such as Visual Analogue Scales (VAS) and Numerical Rating Scales (NRS). Approximately 38.9% of studies included heterogeneous samples of cancer patients, and 66.7% adopted non-pharmacological strategies in experimental group. The most frequently used non-pharmacological strategy was musicotherapy (55.6%). For pharmacological strategies, 61.1% of experimental groups used WHO three-step analgesic ladder and/or analgesic pumps, and 16.7% of control groups employed WHO three-step ladder. More than half of the included studies (66.7%) adopted a combination of pharmacological and non-pharmacological strategies. Dosage and type of analgesic were reported in detail in only one study [25]. Finally, less than half of studies (44.4%) provided care time (mean: 4.63 weeks, median: 4; range: 2–12) and most studies (83.3%) were conducted in general hospital.

**Table 1:** Characteristics of the included studies (*N* = 18)

First Author, Years	Age (Mean)	Subjects n1 + n2	Database	Outcomes	Cancer type	Pharmacological strategies		Non-pharmacological strategies used in experimental group	Care time (week)	Settings
						experimental group	control group			
Li, 2006 [30]	76.26	38 + 38	CNKI/Vip/Wangfang	VAS	Mixed	intravenous analgesic pumps	WHO 3-step ladder	–	4	general hospital
Huang, 2010 [28]	60–88 (72.79)	40 + 80	CNKI/Wanfang	VAS	Mixed	WHO 3-step ladder	WHO 3-step ladder	–	4	primary hospital
Xu, 2014 [35]	58.7	25 + 25	CNKI/Vip/Wangfang	VAS	Mixed	Analgesic drugs	Analgesic drugs	–	3	CTM hospital
Kang, 2015 [29]	52–78 (68.2)	34 + 34	All	VAS	Liver	intravenous analgesic pumps	–	–	–	general hospital
Wu, 2015 [33]	43	74 + 74	Wanfang	NRS	Mixed	WHO 3-step ladder	–	Music and Sport	–	general hospital
Liu, 2016 [31]	55–81 (68.75)	32 + 32	All	VAS	Liver	WHO 3-step ladder	WHO 3-step ladder	Music and Massage	2	general hospital
Su, 2016 [25]	43–78 (72)	63 + 63	All	VAS	Liver	WHO 3-step ladder morphine sulfate (30-60 mg, 1–2/s), nefopam, dolantin	Analgesic drugs	Not in detail	4	general hospital
Fei, 2016 [24]	45–83 (60.47)	26 + 26	CNKI/Vip/Wangfang	VAS	Liver	Analgesic drugs	–	Music and Meridian	–	general hospital
Wu, 2017 [34]	59–80 (69.41)	10 + 10	CBM/Vip/Wangfang	VAS	gastric	intravenous analgesia	–	Acupuncture, Music Massage and Communicate	–	CTM hospital
Luo, 2017 [32]	32–70 (48.9)	25 + 25	All	NRS	Liver	WHO 3-step ladder	–	Acupuncture, Music and Psychotherapy	4	general hospital
Gao, 2017 [26]	28–86 (51.42)	34 + 34	Wanfang	VAS	Mixed	intravenous analgesic pumps	–	Music	–	general hospital
He, 2017 [27]	34–86 (62.13)	40 + 40	All	NRS	Mixed	WHO 3-step ladder pump high-dose morphine	–	Massage and Communicate	–	general hospital
Yao, 2017 [36]	42–80 (60.5)	30 + 30	Wanfang	VAS	Liver	Analgesic drugs	–	Music and Meridian	–	general hospital
Zhang, 2018 [37]	27–82 (63.95)	45 + 45	All	VAS	Lung	Analgesic drugs morphine	–	–	4	general hospital
You, 2018 [38]	50–75 (59.58)	34 + 34	CNKI	NRS	Mixed	Analgesic drugs	Analgesic drugs	Music and Communicate	–	general hospital
Rao, 2018 [39]	33–77 (56.55)	40 + 40	CNKI/Vip/Wangfang	VAS	Liver	WHO 3-step ladder	Analgesic drugs	Music, Communicate and Massage	–	general hospital
Cao, 2018 [40]	38–74 (51.39)	25 + 25	Wanfang	VAS	Liver	Analgesic drugs	–	Music and Communicate	–	general hospital
Yang, 2019 [41]	60–80 (71.25)	50 + 50	CNKI/Wangfang	VAS	Lung	Analgesic drugs	–	–	12	general hospital

Abbreviations: n1 participants in experimental group, n2 participants in control group, *VAS* Visual Analogue Scales, *NRS* Numerical Rating Scales, *CTM* Chinese traditional medicine

### Study quality assessment

Study quality ratings for each criteria of the modified Jadad were indicated in Table 2. Higher scores reflected the better study quality, and the average scores of all studies were above 3 (mean: 3, median: 3; range: 2–4). Six studies were judged to have high quality, and other studies were rated as medium quality.

**Table 2:** Assessment of study quality

Studies	Quality Indicators from the modified Jadad scale					Total score
	A	B	C	D	E	
Li, 2006 [30]	1	1	0	0	1	3
Huang, 2010 [28]	1	0	0	1	1	3
Xu, 2014 [35]	1	1	0	0	0	2
Kang, 2015 [29]	1	1	0	1	1	4
Wu, 2015 [33]	1	0	0	1	0	2
Liu, 2016 [31]	1	0	0	0	1	2
Su, 2016 [25]	1	1	0	1	1	4
Fei, 2016 [24]	1	1	0	0	1	3
Wu, 2017 [34]	1	0	0	1	1	3
Luo, 2017 [32]	1	1	0	1	1	4
Gao, 2017 [26]	1	1	0	1	0	3
He, 2017 [27]	1	1	0	1	1	4
Yao, 2017 [36]	1	0	0	0	1	2
Zhang, 2018 [37]	1	1	0	1	1	4
You, 2018 [38]	1	1	0	1	1	4
Rao, 2018 [39]	1	1	0	0	1	3
Cao, 2018 [40]	1	0	0	0	1	2
Yang, 2019 [41]	1	0	0	0	1	2

Abbreviations: A represents “Was the study described as randomized?” (1: Yes; 0: No); B represents “Was the method of randomization appropriate?” (1: Yes; 0: Not described; −1: No); C represents “Was there a description of withdrawals and dropouts?” (1: Yes; 0: No); D represents “Was there a clear description of the inclusion/exclusion criteria?” (1: Yes; 0: No); E represents “Was the methods of statistical analysis described?” (1: Yes; 0: No)

### Effects of PC on cancer pain

**Fig. 2:**
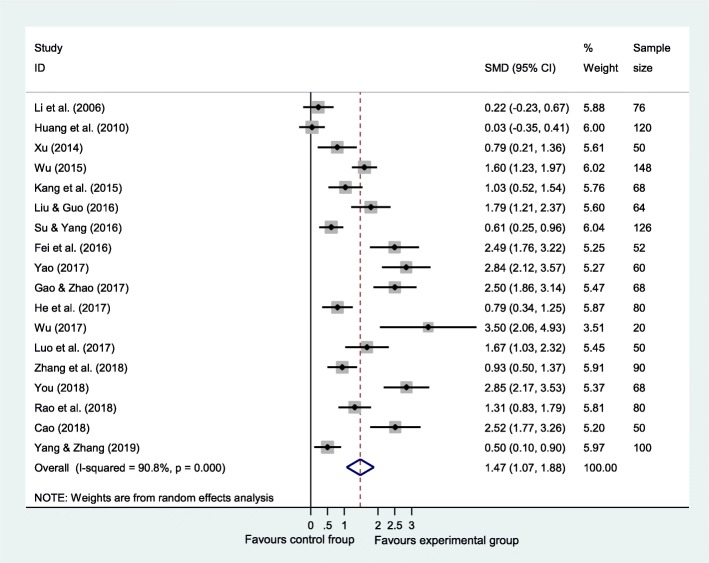
Forest plot of the effect of palliative care on cancer pain. It shows a pooled SMD of 1.475 (95% CI = 1.071–1.878, *p* < 0.001) in random-effect model, indicating that palliative care could alleviate pain among Chinese adults with cancer. Abbreviations: SMD, standardized mean difference.

A pooled random-effect meta-analysis was conducted using data from 18 studies, which estimated the post-test effects of PC on cancer pain compared with care-as-usual control group. As shown in Fig. 2, the random-effect model indicated an overall effect size of 1.475 (95% CI = 1.071–1.878, *p* < 0.001). The heterogeneity analysis (Q = 184.81, *p* < 0.001; I^2^ = 90.8%) revealed that there was a relatively high amount of heterogeneity in our meta-analysis.

### Subgroup analysis

As shown in Table 3, age, pharmacological/non-pharmacological strategies and publication date were the significant sources of heterogeneity to some extent, and their moderating effects were significant for the effect of PC on cancer pain (*p* < 0.01). Effect size was the largest in patients less than 60 years old (SMD = 1.859, 95% CI = 1.348–2.369), but it was the smallest (SMD = 0.348, 95% CI = 0.081–0.616) among patients aged above 70. Compared with pharmacological strategies used in experimental-control group (SMD = 1.054, 95% CI = 0.423–1.686), the effect size was larger than the pharmacological strategies only used in experimental group (SMD = 1.75, 95% CI = 1.255–2.245). Effect size was larger (SMD = 1.954, 95% CI = 1.473–2.435) in the experimental group using non-pharmacological strategies than studies without non-pharmacological strategies (SMD = 0.564, 95% CI = 0.233–0.895). Additionally, effect size was the smallest (SMD = 0.3, 95% CI = − 0.104 to 0.705) for studies published before 2015.

**Table 3:** Effects of palliative care on cancer: subgroup analysis

Subgroup	No.of studies	No.of subjects	SMD	95%CI	Q	I^2^(%)	P^a^
Age (years)							< 0.001
< 60	7	514	1.859***	1.348–2.369	34.01***	82.4	
60–70	7	434	1.781***	1.142–2.420	46.95***	87.2	
> 70	4	422	0.348*	0.081–0.616	5.51	45.6	
Caner type							0.001
Liver	8	550	1.741***	1.177–2.304	55.80^***^	87.5	
Mixed	7	610	1.230**	0.449–1.962	97.31^***^	93.8	
Pharmacological strategies							< 0.001
Used in both groups	7	584	1.054**	0.423–1.686	73.36 ^***^	91.8	
Used in experimental group	11	786	1.750***	1.255–2.245	86.59 ^***^	88.5	
Non-pharmacological strategies							< 0.001
Used in experimental group	12	866	1.954***	1.473–2.435	93.62 ^***^	88.2	
Not used	6	504	0.564**	0.233–0.895	16.19 ^**^	69.1	
Publication date							< 0.001
< 2015	3	246	0.300	−0.104-0.705	4.59	56.4	
2015–2016	5	458	1.461***	0.866–2.055	30.83 ^***^	87.0	
2017	5	278	2.167***	1.235–3.100	36.73 ^***^	89.1	
2018–2019	5	388	1.580***	0.776–2.384	47.95***	91.7	

Abbreviations: *SMD* standardized mean difference

^**^ *p* < 0.01; ^***^ *p* < 0.001

^a^ P of comparison between these subgroups [19], which is akin to analysis of variance. We partition the total variance into variance within groups and variance between groups, and then test these various components of variance for statistical significance, with the last (variance between groups) addressing the hypothesis that effect size differs as function of group membership

## Discussion

We performed strict inclusion criteria and subgroup analysis to reduce the heterogeneity. However, heterogeneity was still relatively high within some subgroups, and the conclusion should be considered with some caution. On the other hand, according to the modified Jadad scale assessing study quality, studies in our meta-analysis with the lack of description about withdrawals/dropouts might weaken the internal validity to some extent.

The present meta-analysis, to our knowledge, was the first to explore the effect and associated moderator variables of PC on pain among Chinese cancer patients, and PC was proven largely effective to relieve pain (SMD = 1.475, 95% CI = 1.071–1.878). The possible explanation to this finding could be attributed to the interface between cancer pain and PC. Contrary to pain traditionally considered as a physiological experience, cancer pain is a complex subjective human experience affected by physical, psychological, social and spiritual components [42], which is of great importance in the care of advanced cancer patients. Correspondently, the aim of PC is to control pain and other symptoms, manage mental, social and spiritual problems, and improve the lives of patients with terminal illness [6], which could capture the complexity of cancer pain.

Age, pharmacological/non-pharmacological strategies and publication date contributed to the significantly substantial heterogeneity to some extent. Compared with other age groups, the effect size was the smallest (SMD = 0.348, 95% CI = 0.081–0.616) among patients aged above 70. Among older cancer patients, there was an attitude of ‘getting on with life’ regardless of pain in comparison with younger counterparts, indicating that older patients revealed acceptance and tolerance of pain to some extent [43]. Several studies also found a relation between more advanced age and less cancer pain [44, 45]. Furthermore, older patients are considered to be more susceptible to opioid-related side effects, and chronic disease treatment might render the pain control more difficult [46]. The opinions mentioned above might lead to that cancer pain were found to be more likely to be unrecognized and undertreated among older patients [46, 47]. As a result, the effect of PC on older patients’ pain were significantly smaller than that on younger patients’ pain in our meta-analysis.

For pain relief, the effects of pharmacological strategies used alone in experimental group were larger than that both used in experimental-control group. The effectiveness of pharmacological approach has been confirmed in the vast majority of patients with cancer pain according to WHO three-step ladder [46, 48, 49]. Additionally, the effect of PC on cancer pain was still significant even though both experimental group and control group adopted pharmacological strategies, which could be explained by the different pharmacological approaches. Several studies suggested better overall pain control and fewer complications with intravenous analgesic pumps than conditional provider-administered analgesia [50, 51]. Another possible explanation might be the non-pharmacological strategies also used in these included studies [25, 31, 38, 39]. RCT found that both non-pharmacological strategies and intravenous analgesic pumps adopted could better improve pain control compared with analgesia pumps adopted alone [52].

Besides of physiological components, other components of human functioning (e.g., personality, mood, behavior, social relations) also play an important role in cancer pain management. By comparison with non-pharmacological strategies not used in the experimental groups, non-pharmacological strategies used in the experimental groups had more beneficial effects on reducing cancer pain. The findings were in line with the literature indicating that in addition to pharmacological strategies, various methods of non-pharmacological treatments, such as psychological, cognitive and behavior therapies, could also relieve cancer pain [13, 53, 54].

With regard to publication date, studies published before 2015 had the smallest effect size of PC on cancer pain [28, 30, 35], mainly because pharmacological strategies were both used in experimental and control group, and non-pharmacological strategies were not used among these studies. It should be noted, however, that because the statistical power of subgroup analysis in this subgroup was limited due to the small number of available studies (*n* = 3), the present result might be overestimated, and clearly there is need for further studies.

### Clinical implications

The present meta-analysis provided several clinical implications. First, PC has long been recognized to provide better pain management and symptom control for patients with advanced illness in developed countries, but aggressive treatment of advanced cancer patients is prevalent in China [55], which poses problems for the development of PC. Based on our findings, PC has been proven to be effective for relieving pain in Chinese cancer patients; second, our findings also provided pharmacological and non-pharmacological guidance in developing optimal approach and appropriate standards of PC in clinical practice. For instance, intravenous analgesic pumps should be adopted for patients with cancer pain at PC units, especially for patients who are ineffectual to conventional pain control. Moreover, non-pharmacological strategies are undoubtedly essential issues in cancer pain management, which should be recommended as pain control methods used concurrently with pharmacological interventions.

## Limitation

Our meta-analysis had several drawbacks that should be taken into consideration in interpreting the findings. First, subgroup analysis was used to explore potential sources of heterogeneity, but substantial heterogeneity of SMD was still observed in both the overall analysis and subgroups analysis (I^2^ > 75%). Thus, we assumed that there were other factors, which are not provided by the included studies, likely influencing heterogeneity; second, as a psychometrically satisfactory instrument for assessment of pain intensity, VAS was used in the included studies, but it cannot provide information about the duration, frequency or interference of pain [56]; third, due to the lack of follow-up results, it is not confirmed whether there were long-term PC effects; fourth, most of the included studies were conducted in general hospitals, which might be difficult to reflect the whole picture of Chinese PC for cancer pain; fifth, further studies need to be conducted to examine whether the findings of our meta-analysis are suitable to other countries; finally, the evaluation of publication bias generally is not useful when less than 20 studies are included in a meta-analysis. Therefore, we did not evaluate the publication bias neither graphically nor using a statistical test in this context due to the small number of included studies (*N* = 18).

## Conclusion

Although there are several limitations (small number of included studies and high heterogeneity) in this meta-analysis, a tentative and preliminary conclusion could be reached that PC was effective for relieving pain in Chinese cancer patients. Besides, medical personnel should pay more attention to the moderating effects of age and pharmacological/non-pharmacological strategies on cancer pain in the process of PC service. However, the findings should be interpreted cautiously, with consideration of high heterogeneity.

## Additional files

Additional file 1: Search strategies for all databases. (PDF 118 kb)

Additional file 2: Selection process of studies for the meta-analysis (international databases). (PDF 19 kb)

## Data Availability

The datasets used and/or analyzed during the current study are available from the corresponding author on reasonable request.

## Abbreviations

CBM: Chinese Biomedical Literature Database
CI: Confidence interval
CNKI: China National Knowledge Infrastructure
NRS: Numerical Rating Scales
PC: Palliative care
RCTs: Randomized controlled trials
SD: Standard deviation
SMD: Standardized mean difference
VAS: Visual Analogue Scales
WHO: World Health Organization
