# Supplementary material for: A systematic review and meta-analysis of randomized controlled trials of palliative care for pain among Chinese adults with cancer
Source: BMC Palliat Care. 2019 Aug 8;18:69. doi: 10.1186/s12904-019-0456-z (PMC6688327; doi:10.1186/s12904-019-0456-z)
Supplement: Supplementary file 1 — Search strategies for all databases. (PDF 118 kb) [file 12904_2019_456_MOESM1_ESM.pdf]

Additional file 1 Search strategies for all databases

Database	Search strategy
Medline /PubMed	(palliative care[MeSH Terms] OR hospice care[MeSH Terms] OR terminal care[MeSH Terms]) AND (neoplasms[MeSH Terms] OR cancer [Title/Abstract] OR neoplasms[Title/Abstract] OR oncology[Title/Abstract]) AND (China[MeSH Terms] OR China or Chinese or Mainland China [Title/Abstract]) And (randomized[Title/Abstract] OR random[Title/Abstract] OR control[Title/Abstract] OR randomly[Title/Abstract]) AND pain [Text word]
SCIE	TS=("palliative care" OR "hospice care" OR "terminal care") AND (TS=neoplasms OR TS=oncology OR TI=cancer OR TI=neoplasms OR TI=oncology) AND (TS=China OR TS=Chinese OR TI=China OR TI=Chinese) AND (TS=random OR TI=randomized OR TI=control OR TI=randomly)
EBSCO	**S1** SU palliative care OR SU hospice care OR TI palliative care OR TI hospice care AND TX ("random allocation" OR "randomized controlled trial*" OR "controlled clinical trial*")<br>**S2** TI China OR TI Chinese OR AB China OR AB Chinese<br>**S3** TI cancer OR TI neoplasms OR TI oncology<br>**S4** **S1** AND **S2** AND **S3**
CNKI/CBM/ Vip/Wanfang	**#1** SU=随机 OR SU=随机分配 OR SU=随机对照 OR SU=对照 OR SU=随机对照试验 OR SU=随机对照研究 OR AB=随机（模糊匹配）<br>**#2** SU=临终挂怀 OR SU=安宁 OR SU=舒缓 OR TI=临终关怀（模糊匹配）<br>**#3** SU=癌症 OR SU=肿瘤 OR TI=癌 OR TI=肿瘤 AND AB=疼（模糊匹配）<br>**#4** **#1** AND **#2** AND **#3**

Abbreviations: CNKI, China National Knowledge Infrastructure; CBM, Chinese Biomedical Literature Database.
