# Supplementary material for: A systematic review and meta-analysis of randomized controlled trials of palliative care for pain among Chinese adults with cancer
Source: BMC Palliat Care. 2019 Aug 8;18:69. doi: 10.1186/s12904-019-0456-z (PMC6688327; doi:10.1186/s12904-019-0456-z)
Supplement: Supplementary file 2 — Selection process of studies for the meta-analysis (international databases). (PDF 19 kb) [file 12904_2019_456_MOESM2_ESM.pdf]

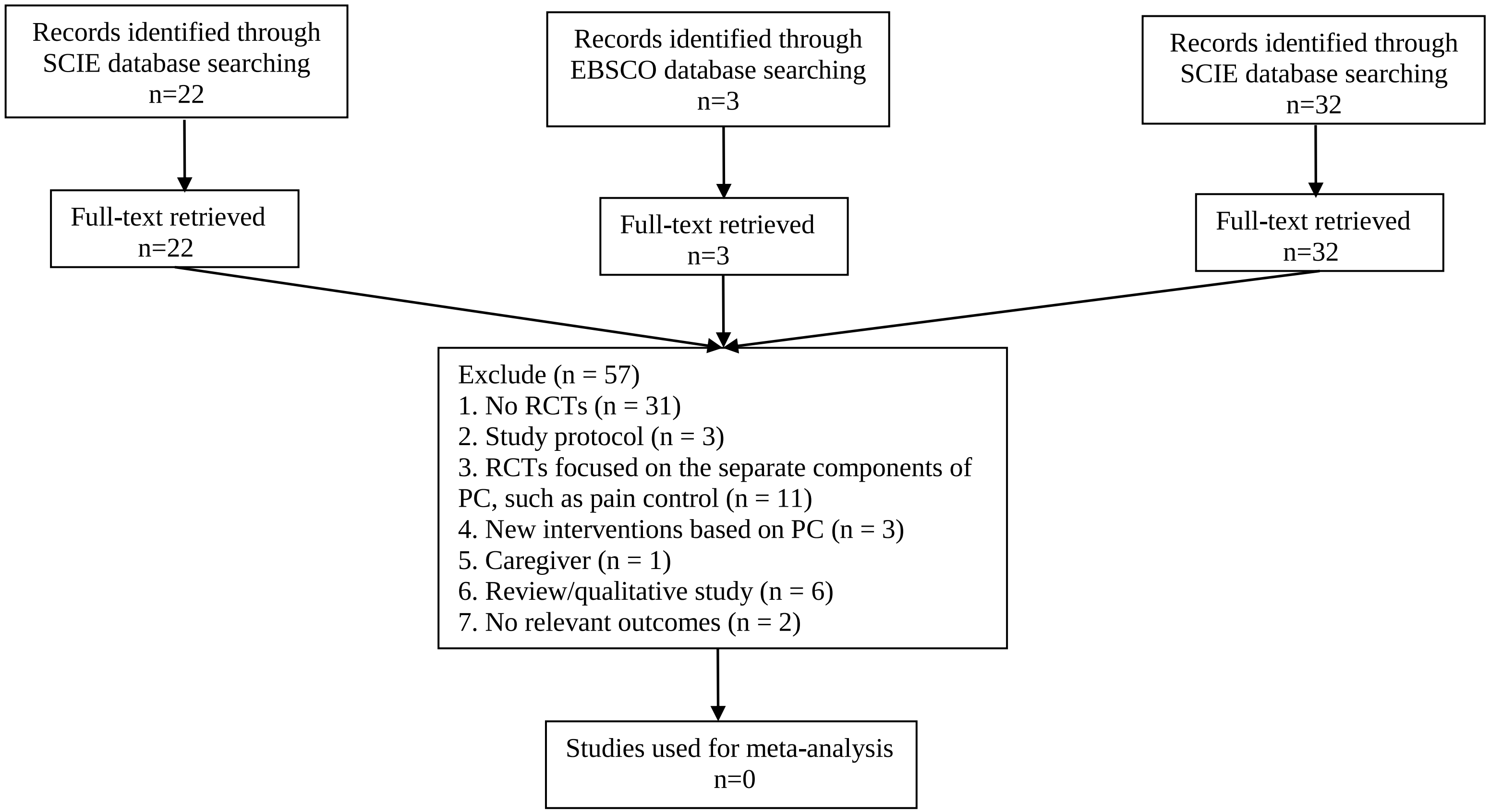

Additional file 2 Selection process of studies for the meta-analysis (international databases)

Abbreviations: RCTs, randomized controlled trials; PC, palliative care.
